# The Department of Modern Nursing

**Published:** 1915-03-13

**Authors:** 


### THE DEPARTMENT OF MODERN NURSING.

### IX.—Guy's Hospital (*continued from page* 496).

### THE PRELIMINARY TRAINING SCHOOL.

#### Lectures and Classes.

Once a week, at 8.15 p.m., first-year probationers attend courses of lectures upon surgery, in the months of April, May, and June; nursing, in the months of July, August, and September; medicine, in the months of October, November, and December.

Weekly classes are held between the lectures, at which probationers produce the notes taken by them, and receive instruction from the sisters.

Second-year probationers attend a course of lectures and practical instruction in pharmacy and dispensing, given by the pharmacist to the hospital, during the months of January, February, and March.

The following prizes are awarded :—

Cazenove Prizes, awarded to probationers who obtain the highest aggregate percentage of marks at the three examinations during their first year : 1st, silver-gilt medal and books; 2nd, silver-gilt medal; 3rd, silver medal.

Keogh Prize (nursing instruments), awarded to the probationer obtaining the highest number of marks in the Surgical Examination.

Hospital Prizes (medical books), awarded to probationers obtaining the highest number of marks in the Medical and Nursing Examinations.

In addition to these lectures and classes, an optional and limited number of senior probationers are coached for massage by qualified sisters, and are allowed extra time off duty for their study in this subject. The hospital pays the fee, and in return the nurse massages any patients who may be receiving this treatment in the wards when she is qualified. There is also a massage department for out-patients. A senior probationer, it should be explained, is one who has passed her examinations in surgery, nursing, and medicine successfully, after her first year's work, and received her "stripes." These consist of a band of white braid worn on both sleeves of the dress just below the elbow and they distinguish the probationer as in her second year. Having passed these examinations, and later the "dispensing" examination, the probationer is free to take up massage should she so desire, as already explained.

#### Time-table for Night Duty.

| | | | |
|---|---|---|---|
| Called ... | ... 8.0 p.m. | In wards ... | Half-hour later. |
| Breakfast ... | ... 8.40 p.m. | Leave wards | 8.0 to 8.30 a.m |
| Prayers ... | ... 9.0 p.m. | Dinner ... | ... 9.0 a.m. |
| In wards ... | ... 9.15 p.m. | Prayers ... | ... 9.30 a.m |
| Night meal— | | Supper ... | ...12.0 noon |
| In Home, 1, 1.30, or 2 a.m. | | In rooms not later than 1.0 p.m. | |

The third year of training at Guy's consists of head nurse's work. If the nurse is considered capable and sufficiently good at the end of two years she receives what are known as her "strings"—*i.e.*, in addition to the regulation "Sister Dora" cap, muslin strings are attached, and a bow worn under the chin which denotes the rank of a third-year or head nurse.

The rules with regard to routine for meals and general regulations must still be observed by the nurse, but in the wards she has to help with the training of probationers and has the general methods of the ward to maintain, and during the absence of the sister she is entirely responsible for everything connected with the management of the ward and the nursing of the patients.

#### Time-table for Head and Staff Nurses.

The time-table for day nurses is the same as that for probationers, except that they have supper half an hour later. The duties of day nurses are as follows :—

In the morning they measure and put up specimens as ordered; see that testing-trays are clean, and reagents ready for use; wash and attend to the most critical cases; give medicines; help to serve the patients' dinner. In the afternoon they see that all is ready for the visit of the staff, whom they take round in the absence of the sister; afterwards they assist in preparing the patients' tea. In the evening they make beds, wash backs, and generally attend to the requirements of the patients, taking care that they are left comfortable for the night. The nurses must also see that the wards, lavatories, etc., are perfectly tidy before they go off duty. The duty of giving medicine and stimulants may be entrusted by the sister to a nurse who has received instruction in dispensing. The nurses are expected, so far as they are able, to teach probationers, and to take care that the work entrusted by them to probationers is efficiently performed. They must immediately report any change they may observe in a patient to the sister, and they must at all times bring to her notice any circumstance which, in their opinion, may prove hurtful to the patients or injurious to the interests of the hospital.

A staff nurse receives a salary of £25 for the first year of service as a staff nurse, £28 for the second year, and £30 for the third and subsequent years. She is required to be a member of the Nurses' League. As an encouragement to thrift, the hospital takes out on her behalf a pension with the Royal National Pension Fund for Nurses for an annuity of £11 5s. at the age of fifty years, the nurse taking out a similar policy for not less than £7 10s. A sister or nurse retires from service at the age of fifty years, but by special resolution of the Court of Governors she may be retained upon the active list during pleasure for a further period not exceeding five years.

In case of sickness a staff nurse will receive full salary during such sickness for a period not exceeding twelve weeks, less (*a*) where the nurse is insured under the National Insurance Act, 1911, a sum equal to the amount to which she is entitled under that Act for sickness benefit, or (*b*) where the nurse has claimed exemption from the Act on the ground of assured private income, a sum equal to one-half of the sickness benefit to which she would have been entitled under the Act had she insured. If at the end of twelve weeks the nurse is still unable to resume her duties, her engagement shall thereupon be deemed to determine at the expiration of such twelve weeks. Any subsequent payment by the hospital to the nurse shall not create a fresh agreement of employment and such payment may be discontinued at any time without liability on the part of the hospital to give any notice.

*Time off Duty.*—This is the same as that for probationers, excepting that the head nurse has a week-end off duty once a month, either Saturday and Sunday or Sunday and Monday, and her annual holiday is one month

